# Evaluation of Carbapenem Use Before and After Implementation of an Antimicrobial Stewardship-Led Carbapenem-Sparing Strategy in a Lebanese Tertiary Hospital: A Retrospective Study

**DOI:** 10.3389/fcimb.2022.729491

**Published:** 2022-03-25

**Authors:** Mira El Masri, Nisrine Haddad, Therese Saad, Nesrine A. Rizk, Ramia Zakhour, Souha S. Kanj, Rony M. Zeenny

**Affiliations:** ^1^ Department of Pharmacy, American University of Beirut Medical Center, Beirut, Lebanon; ^2^ Department of Internal Medicine, American University of Beirut Medical Center, Beirut, Lebanon; ^3^ Department of Pediatrics and Adolescent Medicine, American University of Beirut Medical Center, Beirut, Lebanon

**Keywords:** antimicrobial stewardship (AMS), carbapenem, clinical pharmacy services, infectious diseases, Middle East, Lebanon

## Abstract

**Purpose:**

The use of carbapenem before and after implementation of an antimicrobial stewardship-led carbapenem-sparing strategy at a tertiary care center in Lebanon was evaluated.

**Methods:**

A retrospective, observational chart review was performed on all hospitalized pediatric and adult patients who received carbapenem therapy during January 2019 and January 2020. Patients who started their regimen before January or received carbapenems for less than 24 hours were excluded. Primary outcomes included the appropriateness of physician prescribing patterns and pharmacists’ interventions, as well as appropriateness and response rates of the latter. Secondary outcomes included the carbapenem defined daily dose (DDD) and days of therapy (DOT). Descriptive statistics were used in the analysis and a p-value < 0.05 was considered to be statistically significant.

**Results:**

A total of 157 and 150 patients charts were reviewed in January 2019 and January 2020, respectively. There was no difference in baseline characteristics except for inpatient services and rates of isolated multidrug-resistant organisms. When comparing the two timelines, the appropriateness of physicians’ prescribing patterns increased in terms of empirical therapy, targeted therapy, and duration of therapy but the results were not statistically significant. Pharmacists’ interventions significantly increased with regards to the duration of therapy (p= <0.001), dose adjustment (p<0.001), de-escalation to a narrower spectrum antibiotic (p=0.007), and use of extended infusion (p=0.042). The DDD and DOT were higher for ertapenem and lower for anti-pseudomonal carbapenems in January 2020.

**Conclusion:**

The carbapenem-sparing strategy adopted by the antimicrobial stewardship program contributed to an increase in the number of interventions made by pharmacists on carbapenem therapy, including their appropriateness, and response rate. Despite an improvement in the physician-prescribing patterns, more awareness and education may be needed to achieve a better impact.

## Introduction

Antimicrobial resistance (AMR) is a major worldwide concern affecting global public health. According to the Centers for Disease Control and Prevention (CDC) and the Infectious Disease Society of America (IDSA), more than 2.5 million people are infected with antibiotic-resistant organisms causing at least 34,000 deaths annually and adding more than 2 billion US dollars to direct healthcare costs (CDC, 2019a; Shrestha et al., 2018). Likewise, the World Health Organization (WHO) data revealed an increase in AMR in the Middle East and North Africa region (WHO, 2015). Out of the pathogens with emerging resistance listed in the CDC AMR threats of 2019, carbapenem-resistant *Enterobacterales* (CRE) and carbapenem-resistant *Acinetobacter* (CRA) are urgent threats among patients in the medical facilities^1^. In the United States, It is estimated that around 13,100 and 8,500 healthcare-associated infections (HAI) are caused by CRE and CRA, respectively, each year (CDC, 2019a). Similarly, reports from Médecins Sans Frontieres (MSF) in the Middle East suggested that third-generation cephalosporin and carbapenem resistance among *Enterobacterales* isolates is 86.2% and 4.3% respectively in developing countries like Jordan, Yemen, Iraq, and Syria (Kanapathipillai et al., 2019).

In general, *Enterobacterales* are Gram-negative bacteria that include *Escherichia coli*, *Klebsiella*, and *Enterobacter* species associated with a wide range of community-associated infections as well as HAI (Duin and Doi, 2016). The extensive use of carbapenem in the treatment of infections caused by extended-spectrum beta-lactamase-producing Enterobacterales (ESBL-E) has led to the emergence of CRE as shown in a recent meta-analysis (Codjoe and Donkor, 2017; Loon et al, 2017). The presence of CRE limits treatment options of many severe infections since enzymes produced by these bacteria can hydrolyze many beta-lactams and exhibit other mechanisms of resistance against fluoroquinolones and aminoglycosides (Duin and Doi, 2016). Therapeutic options, such as tigecycline and polymyxins have limited use because of low efficacy, high toxicity, and increasing reports of resistance (Duin et al., 2013; Codjoe and Donkor, 2017). Those are usually used in combination with other agents, and though this strategy has shown a lower mortality rate when compared to monotherapy, the mechanistic basis of synergy is yet to be established (Cui et al, 2017). Another option such as ceftazidime/avibactam has currently been used for CRE but is usually reserved for severe infections due to limited availability and high cost (Sorbera et al., 2014; King et al., 2017). Other novel drugs like meropenem-vaborbactam, cefiderocol, and imipenem-relebactam have shown efficacy against certain CRE strains but are still not available in Lebanon.

In Lebanon, surveillance of AMR has been reported in different clinical and non-clinical settings. The Lebanese Society of Infectious Diseases (LSID) has conducted a study to assess AMR patterns among clinically relevant pathogens between 2011 and 2013 (Chamoun et al., 2016). The ESBL production rate of *Escherichia coli* and *Klebsiella* species was 32.3% and 29.2%, respectively. Similar to international data, the rise in ESBL-producing pathogens has led to an increase in the consumption of carbapenems. As such, lower overall carbapenem susceptibility rates have been reported in a study held between 2015 and 2016, reaching 12% and 70% for *Acinetobacter* species and *Pseudomonas aeruginosa* respectively (Moghnieh et al., 2019). Regarding non-clinical settings, the rise in ESBL and carbapenemase-producing pathogens in water and animals has also been noted (Bayssari et al., 2014; Osman et al., 2019).

As stated by the CDC, 20-50% of all antibiotics prescribed in U.S. acute care hospitals are either unnecessary or inappropriate (CDC, 2019b). Consequently, the IDSA, in cooperation with the Society for Healthcare Epidemiology (SHEA), has issued guidelines for implementing antimicrobial stewardship programs (ASP) (Barlam et al., 2016). The CDC, the American Society of Health-System Pharmacists (ASHP), and Joint Commission International Standards (JCI) have defined the core elements of hospital ASP to optimize safe, judicious, and appropriate use of antimicrobial agents through multidisciplinary efforts (Duin et al., 2013; Barlam et al., 2016; JCI, nd, 2017).

Many studies have addressed the appropriateness of carbapenem use in health care institutions with and without the presence of ASP. For instance, Di Zhang et al. showed that the adequate use of carbapenems was increased after the implementation of an antimicrobial program and that “the irrational use of carbapenems might be a very important factor underlying the development of carbapenem-resistant *Pseudomonas aeruginosa*” (Zhang et al., 2019). In the Middle East, antimicrobial stewardship (AMS) strategies need further development and a better proactive approach as ASP might still be considered as a novel concept (Nasr et al., 2017). A recent survey, conducted by the infectious diseases working group in Arab countries of the Middle East, highlighted the importance of promoting cross-regional collaboration in antimicrobial stewardship. As a result, heterogeneity between countries in awareness of local epidemiology, management of multi-drug resistant organisms, and antimicrobial stewardship practices have been noted (Al Salman et al., 2021).

To our knowledge, the evaluation of carbapenem use before and after implementation of an ASP carbapenem-sparing strategy has not been comprehensively studied in Lebanese hospitals. The only existing data were published by Moghnieh et al. who studied the impact of handshake antimicrobial stewardship on broad-spectrum antibiotic use and proved a significant decrease in imipenem and meropenem use (Moghnieh et al., 2020). In Lebanon, one study showed that the inappropriate use of imipenem-cilastatin at a tertiary care hospital was mostly related to inadequate dose adjustments (Ramadan et al., 2015). According to the surveillance of antimicrobial resistance at the American University of Beirut Medical Center (AUBMC) between June 2018 and June 2019, the rate of CRE has increased in comparison with previous years for *E.coli*, *Klebsiella pneumoniae*, *Citrobacter*, and *Enterobacter* species (AUBMC, 2019). In compliance with JCI standards, and after many years of stewardship efforts starting in 2007, an official ASP was launched at AUBMC in June 2018 with a dedicated stewardship pharmacist to optimize clinical outcomes and minimize the unintended consequences of antimicrobial use. Accordingly, some of the methods included the provision of education along with prospective audit and feedback. In April 2019, the ASP started working on a strategy to optimize carbapenem use. Thus, this study aims to evaluate the use of carbapenems before and after implementation of an ASP-led carbapenem-sparing strategy at AUBMC by comparing physicians’ prescribing patterns and pharmacists’ interventions during January 2019 and January 2020. Other outcomes included the comparison of the carbapenem-specific defined daily dose (DDD) and the days of therapy (DOT) during these two periods.

## Materials and Methods

### Study Design and Population

A retrospective chart review pilot study, evaluating data before and after an intervention, was conducted at AUBMC, a tertiary care 420-bed teaching hospital located in Beirut, encompassing a wide variety of departments and specialized units.

### Inclusion and Exclusion Criteria

The study included all hospitalized pediatric and adult patients who received carbapenem therapy (meropenem, ertapenem, or imipenem/cilastatin) in January 2019 and January 2020. If the patient received multiple courses of carbapenem throughout the same hospital stay, only the first course of treatment was considered. Subsequent carbapenem courses were included for patients with extended hospital stay if they were spaced from the previous ones by at least three months. Patients who were started on a carbapenem regimen before January were not included. Also, those who received carbapenem for less than 24 hours were excluded because their therapy regimen could not have been appropriately assessed in terms of duration of therapy.

### Data Collection

Data was collected retrospectively after patient discharge or discontinuation of therapy. A carbapenem report for both January 2019 and January 2020 was generated and the charts of the patients who received carbapenem therapy during this period were reviewed. The analysis included data collected from the initiation of the first dose of carbapenem until patient discharge. The medical records of admitted patients were reviewed through the hospital’s electronic system. Information on patients receiving carbapenems for treatment of infections in all departments was incorporated using a data collection sheet that included the following: demographic data, past medical history, ID team on board, medications received during hospitalization, type of infection, bacterial culture, and sensitivity results, carbapenem dosing regimen, duration of treatment (including start and end date), serum creatinine, and calculated creatinine clearance according to the Cockcroft–Gault equation in adults and the Schwartz equation in pediatrics. Information about pharmacists’ interventions was also included and divided based on the subtypes of AMS interventions.

### Interventions

In April 2019, the ASP at AUBMC launched a carbapenem-sparing strategy. This strategy initially started with assessing the appropriateness of carbapenem use, which was later presented to the infectious diseases’ (ID) division and ASP committee. The ASP team prospectively reviewed active carbapenem orders and intervened accordingly. As part of this study and in cooperation with the ASP, targeted education sessions focusing on Gram-negative resistance and appropriateness of carbapenem use were provided to both prescribing physicians and pharmacists in December 2019. Each department received one session based on their role in ASP.

### Study Outcomes

Primary outcomes included the appropriateness of physicians’ prescribing patterns as well as the subtypes, appropriateness, and response rates of pharmacists’ interventions. The appropriateness of carbapenem prescribing was assessed before, at, and after 48 hours of antibiotic initiation based on three categories: (i) indication (both empirical and targeted), (ii) dosing, and (iii) duration. The appropriateness of empirical use was assessed based on internally developed algorithms (**Appendix A**) that were approved by the ASP. The algorithms were designed using several references, such as the IDSA guidelines, Surviving Sepsis Campaign guidelines, and the ASHP Pharmacist Guide to Antimicrobial Therapy and Stewardship (Wieczorkiewicz and Sincak, 2016). They also included risk factors for multi-drug resistant organisms (MDRO) based on the type of infection (**Appendix B**). As such, the empirical carbapenem treatment was considered appropriate if the patient had any MDRO risk factors. If the culture did not show any organism, the assessment was based on other criteria such as imaging findings, hematology lab values, and patient’s overall clinical stability (**Appendix A**). The targeted use of carbapenem was considered appropriate if the microorganism was only susceptible to carbapenems and de-escalation was not possible. The dose and duration were deemed appropriate if they were within the recommended range for the specific indication as recommended per the IDSA guidelines.

Antimicrobial stewardship pharmacists' interventions consisted of five major categories: (i) duration of antibiotic therapy, (ii) de-escalation to a narrower-spectrum antibiotic, (iii) dose adjustment, (iv) drug regimen modification because of bug-drug mismatch, (v) and limiting duplicate coverage of antibiotics.

Secondary outcomes included comparing the DDD and the DOT in the two study screening periods. As per the WHO, the DDD is defined as the average maintenance dose per day for a drug used for its main indication (WHO, 2019). The DOT is the aggregate sum of days for which an antibiotic is administered. The DDD and DOT were respectively calculated per 100 bed-days and 1000 patient-days.

### Statistical Analysis

Statistical analyses were performed using the Statistical Package for the Social Sciences for Windows, Version 23.0 (SPSS Inc. - IBM Corp., Armonk, NY, USA) (IBM, 2015). Descriptive statistics were calculated. Means and standard deviations were reported for continuous variables. Categorical variables were assessed and described as frequency and percentage. The associations between categorical variables were evaluated using Pearson χ2 test or Fisher’s exact test. A p-value< 0.05 was considered to be statistically significant.

### Ethical Consideration

The study protocols were approved by the Institutional Review Board (IRB) prior to the start of the study. Per local policies, written patient consent was not required.

## Results

### Baseline Characteristics and Carbapenem Therapy Distribution

A total of 307 patients were included in this study (pre-implementation phase, January 2019 n= 157; post-implementation phase, January 2020, n=150). Only 47 (15%) of enrolled patients were from the pediatric population. Baseline characteristics were similar between both groups except for the fact that more patients were admitted to the intensive care unit in January 2020 (36%) in comparison with January 2019 (19.7%) (p-value =0.001) (**Table 1**). In addition, more patients in January 2020 had recent hospitalization within the past 90 days (57.8% versus 42.2%; p-value=0.018) and a history of recurrent urinary tract infections (15.3% versus 5.1%; p-value=0.004). Concerning the site of infections, there was no statistically significant difference between January 2019 and January 2020 groups and the three most common sites of infection were the lungs (30% versus 27%), urinary tract (28% versus 27%), and bloodstream (16% versus 12%). In January 2020, the rate of empirical use of carbapenem therapy had increased in comparison with January 2019 (85% versus 82%, p-value=0.537) but, in parallel, a significant increase in targeted therapy was noted (23.3% versus 11.5%; p-value=0.007).

**Table 1:** Demographic and clinical variables in study populations.

Variables	January 2019 (n=157)	January 2020 (n=150)	p-Value
Mean ± S.D. age adults, yr (n= 260)	64.1 ± 20.8	68 ± 18.2	0.1
Mean ± S.D. age pediatrics, yr (n= 47)	6.9 ± 6.0	8.81 ± 5.5	0.3
Female, no. (%)	73 (46.5)	71 (47.3)	
Mean ± S.D. BMI (kg/m2)	26 ± 6.5	26.3 ± 6.78	0.814
Service			0.001
Internal Medicine, no. (%)	97 (61.8)	62 (47.1)	
Surgery, no. (%)	18 (11.5)	16 (10.7)	
Intensive Care Unit, no. (%)	31 (19.7)	54 (36)	
Pediatrics, no. (%)	11 (7)	18 (12)	
Mean ± S.D. length of stay, no. (%)	23 (± 37)	18.9 ( ± 25)	0.23
Infectious Diseases Team on Board, no. (%)	136 (86.6)	129 (86)	1
Past Medical History			
Asthma, no. (%)	1 (0.6)	3 (2)	0.361
Atrial fibrillation, no. (%)	17 (10.8)	22 (14.7)	0.392
Cancer, no. (%)	65 (41.4)	70 (46.7)	0.36
Congestive Heart Failure, no. (%)	16 (10.2)	21 (14)	0.381
Chronic Obstructive Pulmonary Disease, no. (%)	12 (7.6)	11 (7.3)	1
Chronic Kidney Diseases, no. (%)	25 (15.9)	23 (15.3)	1
Hypertension, no. (%)	52 (33.1)	51 (34)	0.904
Dyslipidemia, no. (%)	25 (15.9)	21 (14)	0.749
Stroke, no. (%)	7 (4.5)	9 (6)	0.613
MDRO Risk factor			
Recent Antibiotic use within the past 90 days, no. (%)	66 (42)	55 (36.7)	0.352
Recent Hospitalization within the past 90 days, no. (%)	49 (42.2)	67 (57.8)	0.018
History of recurrent UTI, no. (%)	8 (5.1)	23 (15.3)	0.004
MASCC score < 21, no. (%)	10 (6.4)	6 (4)	0.444
History of *ESBL*, no. (%)	13 (8.3)	11 (7.3)	0.833
History of *CRE*, no. (%)	1 (0.6)	2 (1.3)	0.615
History of *E.coli* MDR, no. (%)	8 (5.1)	2 (1.3)	0.105

S.D., standard deviation; BMI, body mass index; MDRO, multi-drug resistant organisms; UTI, urinary tract infection; MASCC, Multinational Association for Supportive Care in Cancer; ESBL, Extended Spectrum Beta-Lactamase; CRE, carbapenem-resistant Enterobacterales; E. coli, Escherichia coli.

### Primary Outcomes: Appropriateness of Physicians’ Prescribing Patterns

The percentages of appropriateness in empirical use before 48 hours (92.9% versus 86.4%; p-value= 0.104) and after 48 hours from culture results (78.9% versus 67.1%; p-value=0.107) and in targeted use (91.4% versus 88.9%; p-value=1) were higher in January 2020 in comparison with January 2019 but the results were not statistically significant (**Table 2**). Similarly, the numbers were in favor of January 2020 concerning the duration of therapy and de-escalation without statistical significance (**Table 2**). The mean duration of carbapenem therapy in January 2019 and 2020 was 4.8 days and 4.7 days, respectively. On the other hand, lower percentages of appropriateness were seen in January 2020 in terms of prophylactic therapy, and the choice of dosing regimens, but no statistically significant differences were detected (**Table 2**).

**Table 2:** Percentage of appropriateness of physicians’ prescribing patterns in terms of carbapenem therapy in January 2019 and January 2020.

	No. (%) Patients		
Variable	January 2019	January 2020	p-value
**Empirical therapy < 48 hours**	*n =* 132	*n =* 127	
Appropriate empirical <48 hours	114 (86.4)	118 (92.9)	0.104
**Empirical therapy ≥ 48 hours**	*n =* 79	*n =* 76	
Appropriate empirical ≥ 48 hours	53 (67.1)	60 (78.9)	0.107
**Targeted therapy**	*n =* 18	*n =* 35	
Appropriate targeted	16 (88.9)	32 (91.4)	1
**Prophylaxis**	*n =* 14	*n =* 11	
Appropriate prophylaxis	5 (35.7%)	3 (27.3)	0.695
**Dosing**	*n =* 157	*n =* 150	
Appropriate dosing	119 (75.8)	103 (68.7)	0.202
**Subsequent dosing/frequency**	*n =* 23	*n =* 11	
Appropriate subsequent dosing/frequency	9 (39.1)	2 (18.2)	0.271
**Duration**	*n =* 154	*n =* 147	
Appropriate duration	99 (64.3)	106 (72.1)	0.174
**De-escalation**	*n =* 37	*n =* 36	
Appropriate de-escalation	30 (81.1)	33 (91.7)	0.308

### Primary Outcomes: Pharmacists’ Interventions: Subtypes, Appropriateness and Response Rate

The total number of pharmacists’ interventions increased from 26.8% in January 2019 to 60.9% in January 2020 and the mean number of interventions made per patient significantly improved from 0.23 (± 0.465) to 0.69 ( ± 0.743) (p-value < 0.001) (**Table 3**). There was a significant rise in all interventions’ subtypes in the post-implementation phase versus the pre-implementation phase except for limiting duplicate coverage and bug-drug mismatch. As for the appropriateness of the interventions, a significant increase was only seen in dose adjustment when comparing January 2020 with January 2019 (100% versus 82.4% respectively; p-value: 0.02) (**Table 3**). In addition, the rate of accepted interventions for de-escalating to a narrower spectrum antibiotic was significantly higher in January 2020 versus January 2019 (90% versus 33.3%; p-value: 0.004) (**Table 3**).

**Table 3:** Pharmacists’ interventions: subtypes, appropriateness, and response rate in January 2019 and January 2020.

	No. (%) Interventions		
Variable	January 2019	January 2020	p-Value
**Duration of therapy**	*n =* 30	*n =* 30	
Number of interventions	7 (23.3)	25 (83.3)	<0.001
Appropriateness	6 (85.7)	25 (100)	0.219
Response Rate	5 (71.4)	22 (88)	0.296
**De-escalation to a narrower antibiotic**	*n =* 35	*n =* 33	
Number of interventions	9 (25.7)	20 (60.6)	0.007
Appropriateness	9 (100)	20 (100)	
Response Rate	3 (33.3)	18 (90)	0.004
**Dose adjustment**	*n =* 46	*n =* 47	
Number of interventions	17 (37)	41 (87.2)	<0.001
Appropriateness	14 (82.4)	41 (100)	0.022
Response Rate	15 (88.2)	40 (97.2)	0.203
**Extended infusion**	*n =* 17	*n =* 37	
Number of interventions	1 (5.9)	13 (35.1)	0.042
Appropriateness	1 (100)	13 (100)	
Response Rate	0 (0)	12 (92.3)	0.143
**Limit duplicate coverage**	*n =* 14	*n =* 19	
Number of interventions	2 (14.3)	2 (10.5)	1
Appropriateness	2 (100)	2 (100)	
Response Rate	1 (50)	2 (100)	1
**Bug-drug mismatch**	*n =* 2	*n =* 2	
Number of interventions	2 (100)	2 (100)	
Appropriateness	0	0	
Response Rate	0	0	

### Secondary Outcomes

The DDD per 100 bed days of all carbapenems was higher in January 2020 (8.6) versus January 2019 (8.1) (**Table 4**). However, in January 2020, the DDD was lower for the antipseudomonal carbapenems and higher for ertapenem. Similarly, DOT per 1000 patient days of the antipseudomonal carbapenem was lower in January 2020 whereas the DOT of ertapenem was higher (**Table 4**).

**Table 4:** Carbapenem-specific defined daily doses (DDD) And days of therapy (DOT) in January 2019 and January 2020.

Variable	January 2019	January 2020
**Defined Daily Doses (g) per 100 bed-days**		
All Carbapenems	8.3	8.6
Combined Antipseudomonal Carbapenems*	6.7	6.1
Non- antipseudomonal Carbapenem	1.5	2.5
**Days of Therapy (days) per 1000 patient-days**		
All Carbapenems	94.3	100.9
Combined Antipseudomonal Carbapenems*	81.3	80.6
Non- antipseudomonal Carbapenem	14.3	22.7

*Meropenem and imipenem/cilastatin.

## Discussion

In this study, we evaluated the impact of a carbapenem-sparing strategy on physicians’ prescribing patterns and pharmacists’ interventions. In an era of increased carbapenem resistance and challenges facing severe gram-negative infections, this strategy is of high importance to promote optimal carbapenem use (Wilson, 2017; Peri et al., 2019). In our study, the rise in empirical use of carbapenems in January 2020 may be partially justified by an increase in the number of patients admitted to the critical care unit. Those patients had more MDROs risk factors which made them candidates for empirical carbapenem therapy. In addition, if stricter criteria were made on the selection of empirical antibiotic use, more significant results could have been reached in this outcome. However, it is worth mentioning that, due to the high rates of ESBL in Lebanon, there is a physician tendency to start a carbapenem instead of another antipseudomonal agent (such as cefepime), if the patient has risk factors of acquiring multi-drug resistant organisms. Accordingly, the appropriateness of physicians’ prescribing patterns improved only numerically without showing any statistical significance. On the other hand, there was an increase in the inappropriateness of carbapenem use in terms of prophylactic use and choice of dosing regimens in the post-implementation phase. This could be explained by the fact that educational sessions that were provided excluded the surgical department, and, physicians at our institution usually tend to depend on clinical pharmacists’ input for dose adjustments of medication. Therefore, more awareness needs to be provided regarding these two areas. In addition to that, a stricter surgical prophylaxis policy must be implemented at our institution with audits and feedback.

In comparison with another study evaluating the use of carbapenem therapy at a tertiary care Chinese hospital, a point score system was adopted to assess the adequacy of the therapeutic regimens and a significant increase in the rational use of carbapenem was seen during three subsequent stages divided between years 2011 to 2017 post-implementation (Zhang et al., 2019). In our study, we did not develop a validated tool for assessment, but we internally developed algorithms assessing therapy appropriateness on three different levels: empirical, targeted, and surgical prophylaxis therapy. A point score system could have been an option but assigning a different number to each item based on their relative impact on the whole therapeutic regimen may lead to subjectivity. Thus, algorithms were developed as guidance materials to reach adequate and objective assessment. Furthermore, it is essential to note that carbapenem-sparing strategy led by ASP was launched in April 2019, only 8 months before the post-intervention period of January 2020. Thus, the establishment of this strategy still needs more time to better assess the program’s effect on the prescribing patterns. In parallel with other studies, Seah et al. summarized the main reasons for inappropriate use of carbapenems necessitating ASP interventions. These reasons include prolonged duration of use, wrong dose, and inappropriate empirical and targeted therapy. The same study has shown an improvement in carbapenem prescribing 2.5 years after ASP implementation (Seah et al., 2017).

Pharmacists have a big role in highlighting the optimal use of antimicrobial agents by promoting the best evidence-based practice and their interventions can have a significant impact on judicious carbapenem use (ASHP, 2010). In our study, a significant improvement was noted in the post-implementation phase as more pharmacy interventions were done with regards to the duration of therapy, de-escalating to a narrower antibiotic, and dose adjustment.

Interventions related to limiting duplicate coverage did not improve in January 2020. Opportunities to limit coverage mostly consisted of a prolonged unwarranted dual antipseudomonal coverage usually in the context of febrile neutropenia in the pediatric oncology population. Therefore, more effort is needed in setting clear guidelines for the management of febrile neutropenia in the pediatric population to avoid such prolonged double coverage.

More interventions were done on therapy de-escalation, and these were accompanied by a higher physician acceptance rate. This might explain the significant increase in targeted therapy in January 2020 whereby ertapenem was chosen over meropenem in the case of ESBL identification. Our results were consistent with those of another study where a significant rise in acceptance rates for de-escalation of carbapenems was seen after 2.5 years of ASP initiation (Seah et al., 2017). De-escalation efforts were also reflected in the DDD and DOT, where higher consumption of ertapenem accompanied by lower antipseudomonal consumption was recorded in January 2020. The overall use of carbapenems did not decrease between the pre-and post-implementation phase of the carbapenem-sparing strategy which could be justified by the short period since implementation and the opposite numerical trend between ertapenem and other antipseudomonal carbapenems. Comparably, in a study looking at the impact of post-prescription review and feedback on carbapenem DOT, a significant decrease in DOT was seen during the third phase of an 8-year ASP strategy (Akazawa et al., 2019).

Several strengths exist in our study. To the best of our knowledge, this is the first local study evaluating the use of carbapenems before and after implementation of ASP in terms of prescribing patterns and pharmacy interventions in both adult and pediatric populations. Second, information was retrieved from EPIC Healthcare System which guaranteed their accuracy. Third, significance was seen only a few months after ASP carbapenem-sparing strategy implementation, which was rather a quick result. Nevertheless, some limitations exist in this project. The study is a single-center observational study; thus, the results of our outcomes cannot be generalized to other institutions whereby different ASP implementation and practices may exist. Also, a relatively small sample size of patients was included since only one month before and after establishing carbapenem-sparing strategy was considered. Accordingly, more significant results could have been reached if the timeframe of the study was longer. Also, some carbapenem regimens were started during the weekend or on holidays where ASP and clinical pharmacists’ activities are limited. Therefore, some interventions may have been missed. Finally, this study did not evaluate the impact of ASP on patient outcomes nor resistance patterns like other studies showing that ASP interventions led to a significant decrease in carbapenem-resistant *Pseudomonas aeruginosa* and did not assess patient safety while reducing carbapenem consumption (Hagiwara et al., 2018; Peri et al., 2019). However, a recent study at our center showed that the control of CRA spread was only achieved when infection control practices were combined with stewardship efforts and restriction of carbapenem use (Rizk et al., 2021). The latter demonstrated a tremendous decrease in CRA colonization pressure per 1000 patient-days in the intensive care units from 210 in the first quarter of 2019 to 0 in the first quarter of 2020 (Rizk et al., 2021).

## Conclusion

The post-implementation phase of an ASP-led carbapenem-sparing strategy was characterized by a significant increase in the use of carbapenems targeted therapy, in the number of antimicrobial stewardship interventions made by pharmacists, and in the acceptance rate with regards to de-escalation to a narrower antibiotic. Despite the overall numerical improvement in physician-prescribing patterns, more awareness and education are still needed especially in terms of carbapenem dosing regimens and appropriate use in surgical prophylaxis. Future studies should include an evaluation of the impact of a carbapenem-sparing strategy on patient-related outcomes and resistance patterns.

## Data Availability Statement

The raw data supporting the conclusions of this article will be made available by the authors, without undue reservation.

## Ethics Statement

The studies involving human participants were reviewed and approved by American University of Beirut. Written informed consent from the participants’ legal guardian/next of kin was not required to participate in this study in accordance with the national legislation and the institutional requirements.

## Author Contributions

NH, TS, RMZ, and NR designed the study. MM, NH, TS, and RMZ drafted and reviewed the questionnaire. RMZ carried out the analysis, and interpreted the results. MM drafted the initial draft of the manuscript. NR, NH, TS, RZ, and SK revised and edited the article. All authors reviewed and approved the final version of the manuscript.

## Conflict of Interest

The authors declare that the research was conducted in the absence of any commercial or financial relationships that could be construed as a potential conflict of interest.

## Publisher’s Note

All claims expressed in this article are solely those of the authors and do not necessarily represent those of their affiliated organizations, or those of the publisher, the editors and the reviewers. Any product that may be evaluated in this article, or claim that may be made by its manufacturer, is not guaranteed or endorsed by the publisher.

## References

[B1] Akazawa T. Kusama Y. Fukuda H. Kayoka H. Kutsuna S. Moriyama Y.. (2019). Eight-Year Experience of Antimicrobial Stewardship Program and the Trend of Carbapenem Use at a Tertiary Acute-Care Hospital in Japan—The Impact of Postprescription Review and Feedback. Open Forum Infect. Diseases 6 (10),e1–6. doi: 10.1093/ofid/ofz389 PMC6790398 31660352

[B2] Al Salman J. Al Dabal L. Bassetti M. Alfouzan W. Maslamani M. Alraddadi B.. (2021). Promoting Cross-Regional Collaboration in Antimicrobial Stewardship: Findings of an Infectious Diseases Working Group Survey in Arab Countries of the Middle East. J. Infect Public Health 14 (7), 978–984. doi: 10.1016/j.jiph.2021.04.009 34130122

[B3] AUBMC. (2019). Antimicrobial Resistance of Pathogens at the American University of Beirut Medical Center (AUBMC).

[B4] ASHP. (2010). ASHP Statement on the Pharmacist’s Role in Antimicrobial Stewardship and Infection Prevention and Control. Am. J. Health-System Pharm. 67 (7), 575–577. doi: 10.2146/sp100001 20237387

[B5] Barlam T. F. Cosgrove S. E. Abbo L. M. Macdoughall C. Schuetz A. N. Septimus E. J.. (2016). Implementing an Antibiotic Stewardship Program: Guidelines by the Infectious Diseases Society of America and the Society for Healthcare Epidemiology of America. Clin. Infect. Diseases. 62 (10), e51–e77. doi: 10.1093/cid/ciw118 27080992 PMC5006285

[B6] Bayssari C. A. Dabboussi F. Hamze M. Rolain J.-M. (2014). Emergence of Carbapenemase-Producing Pseudomonas Aeruginosa and Acinetobacter Baumannii in Livestock Animals in Lebanon. J. Antimicrobial Chemother 70 (3), 950–951. doi: 10.1093/jac/dku469 25406297

[B7] CDC (2019a). Antibiotic Resistance Threats in the United States (Atlanta, GA: U.S. Department of Health and Human Services, CDC).

[B8] CDC (2019b) Core Elements of Hospital Antibiotic Stewardship Programs (Atlanta, GA: US Department of Health and Human Services, CDC) (Accessed November 19,2019).

[B9] Chamoun K. Farah M. Araj G. Daoud Z. Moghnieh R. Salameh P.. (2016). Surveillance of Antimicrobial Resistance in Lebanese Hospitals: Retrospective Nationwide Compiled Data. Int. J. Infect. Diseases 46, 64–70. doi: 10.1016/j.ijid.2016.03.010 26996458

[B10] Cui X. Zhang H. Du H. (2019). Carbapenemases in Enterobacteriaceae: Detection and Antimicrobial Therapy. Front. Microbiol. 10. doi: 10.3389/fmicb.2019.01823 PMC6710837 31481937

[B11] Codjoe F. Donkor E. (2017). Carbapenem Resistance: A Review. Med. Sci. 6 (1), 1. doi: 10.3390/medsci6010001 PMC5872158 29267233

[B12] Duin D. V. Doi Y. (2016). The Global Epidemiology of Carbapenemase-Producing Enterobacteriaceae. Virulence 8 (4), 460–469. doi: 10.1080/21505594.2016.1222343 27593176 PMC5477705

[B13] Duin D. V. Kaye K. S. Neuner E. A. Bonomo R. A. (2013). Carbapenem-Resistant Enterobacteriaceae: A Review of Treatment and Outcomes. Diagn. Microbiol. Infect. Dis 75 (2), 115–120. doi: 10.1016/j.diagmicrobio.2012.11.009- 23290507 PMC3947910

[B14] Hagiwara D. Sato K. Miyazaki M. Kamada M. Moriwaki N. Nakano T.. (2018). The Impact of Earlier Intervention by an Antimicrobial Stewardship Team for Specific Antimicrobials in a Single Weekly Intervention. Int. J. Infect. Dis. 77, 34–39. doi: 10.1016/j.ijid.2018.09.025 30292892

[B15] IBM Corp (2015). IBM SPSS Statistics for Windows, Version 23.0 (Armonk, NY: IBM Corp).

[B16] JCI 6th Edition in Depth The Importance of Antibiotic Stewardship. (n.d.). (2017). Available at: https://www.jointcommissioninternational.org/standards/hospital-standards-communication-center/6th-edition-in-depth-the-importance-of-antibiotic-stewardship (Accessed 5 October 2019).

[B17] Kanapathipillai R. Malou N. Hopman J. Bowman C. Yousef N. Michel J.. (2019). Antibiotic Resistance in Conflict Settings: Lessons Learned in the Middle East. JAC-Antimicrobial Resistance 1(1), e1–e3. doi: 10.1093/jacamr/dlz002 PMC8210113 34222876

[B18] King M. Heil E. Kuriakose S. Bias T. Huang V. El-Beyrouty C.. (2017). Multicenter Study of Outcomes With Ceftazidime-Avibactam in Patients With Carbapenem-Resistant Enterobacteriaceae Infections. Antimicrobial Agents Chemother 61 (7), e00449-17. doi: 10.1128/aac.00449-17 PMC5487633 28483952

[B19] Loon K. Holt A. Vos M. (2017). A Systematic Review and Meta-Analyses of the Clinical Epidemiology of Carbapenem-Resistant Enterobacteriaceae. Antimicrob. Agents Chemother. 62 (1), e01730–17. doi: 10.1128/aac.01730-17 PMC5740327 29038269

[B20] Moghnieh R. Araj G. F. Awad L. Daoud Z. Mokhbat J. Jisr T.. (2019). A Compilation of Antimicrobial Susceptibility Data From a Network of 13 Lebanese Hospitals Reflecting the National Situation During 2015–2016. Antimicrobial Resistance Infect Control 8, 41. doi: 10.1186/s13756-019-0487-5 PMC6381724 30828445

[B21] Moghnieh R. Awad L. Abdallah D. Jadayel M. Sinno L. Tamim H.. (2020). Effect of a “Handshake” Stewardship Program Versus a Formulary Restriction Policy on High-End Antibiotic Use, Expenditure, Antibiotic Resistance, and Patient Outcome. J. Chemother 32 (7), 368–384. doi: 10.1080/1120009x.2020.1755589 32364030

[B22] Nasr Z. Paravattil B. Wilby K. (2017). The Impact of Antimicrobial Stewardship Strategies on on Antibiotic Appropriateness and Prescribing Behaviours in Selected Countries in the Middle East: A Systematic Review. Eastern Mediterranean Health J. 23 (6), E430–E440. 10.26719/2017.23.6.430 28836656

[B23] Osman M. Mir H. A. Rafei R. Dabboussi F. Madec J. Haenni M.. (2019). Epidemiology of Antimicrobial Resistance in Lebanese Extra-Hospital Settings: An Overview. J. Global Antimicrobial Resistance 17, 123–129. doi: 10.1016/j.jgar.2018.11.019 30553113

[B24] Peri A. M. Doi Y. Potoski B. A. Harris P. N. A. Paterson D. L. Righi E. (2019). Antimicrobial Treatment Challenges in the Era of Carbapenem Resistance. Diagn. Microbiol. Infect. Dis. 94 (4), 413–425. doi: 10.1016/j.diagmicrobio.2019.01.020 30905487

[B25] Ramadan W. Kabbara W. Nawas G. (2015). Evaluation of the Appropriateness of Imipenem/Cilastatin Prescription and Dosing in a Tertiary Care Hospital. Infect Drug Resistance 2015, 31. doi: 10.2147/idr.s78633 PMC4378284 25848308

[B26] Rizk N. Haddad N. Zahreddine N. Zeenny R. M. Ahmadieh R. Kanj S.. (2021). “Carbapenem-Sparing Strategy, Carbapenem Consumption, and Acenitobacter Colonization Pressure in a Lebanese Tertiary-Care Center in Beirut,” In Poster Presented at the 31st European Congress of Clinical Microbiology & Infectious Diseases.

[B27] Seah V. X. F. Ong R. Y. L. Lim A. S. Y. Chong C. Y. Tan N. W. H. Thoon K. C. (2017). Impact of a Carbapenem Antimicrobial Stewardship Program on Patient Outcomes. Antimicrobial Agents Chemother 61 (9), e00736–17. doi: 10.1128/aac.00736-17 PMC5571328 28717037

[B28] Shrestha P. Cooper B. S. Coast J. Oppong R. Thi Thuy N. D. Phodha T.. (2018). Enumerating the Economic Cost of Antimicrobial Resistance Per Antibiotic Consumed to Inform the Evaluation of Interventions Affecting Their Use. Antimicrob. Resist. Infect. Control 7, 98. doi: 10.1186/s13756-018-0384-3 30116525 PMC6085682

[B29] Sorbera M. Chung E. Ho C. W. Marzella N. (2014). Ceftolozane/Tazobactam: A New Option in the Treatment of Complicated Gram-Negative Infections. P T 39 (12), 825–832. 10. 25516692 PMC4264669

[B30] WHO (2015). Worldwide Country Situation Analysis: Response to Antimicrobial Resistance. Available at: https://www.who.int/drugresistance/documents/situationanalysis/en/ (Accessed 13 September 2019).

[B31] WHO (2019). Antimicrobial Stewardship Programmes in Health-Care Facilities in Lowand Middle-Income Countries. A Practical Toolkit (Geneva: World Health Organization) (Accessed November 19, 2019).

[B32] Wieczorkiewicz S. M. Sincak C. A. (2016). The Pharmacist’s Guide to Antimicrobial Therapy and Stewardship (Bethesda MD, USA: American Society of Health-System Pharmacists). Available at: https://publications.ashp.org/view/book/9781585285204/978158. Retrieved September 2019.

[B33] Wilson A. P. R. (2017). Sparing Carbapenem Usage. J. Antimicrob. Chemother. 72 (9), 2410–2417. doi: 10.1093/jac/dkx181 28637307

[B34] Zhang D. Cui K. Lu W. Bai H. Zhai Y. Hu S.. (2019). Evaluation of Carbapenem Use in a Tertiary Hospital: Antimicrobial Stewardship Urgently Needed. Antimicrobial Resistance Infect Control 8 (1), e01730–17. doi: 10.1186/s13756-018-0449-3 PMC6322243 30627429

