# Supplementary material for: Evaluation of Carbapenem Use Before and After Implementation of an Antimicrobial Stewardship-Led Carbapenem-Sparing Strategy in a Lebanese Tertiary Hospital: A Retrospective Study
Source: Front Cell Infect Microbiol. 2022 Mar 25;12:729491. doi: 10.3389/fcimb.2022.729491 (PMC8990088; doi:10.3389/fcimb.2022.729491)
Supplement: Supplementary file 1 [file DataSheet_1.pdf]

Empiric Carbapenem Therapy Algorithm

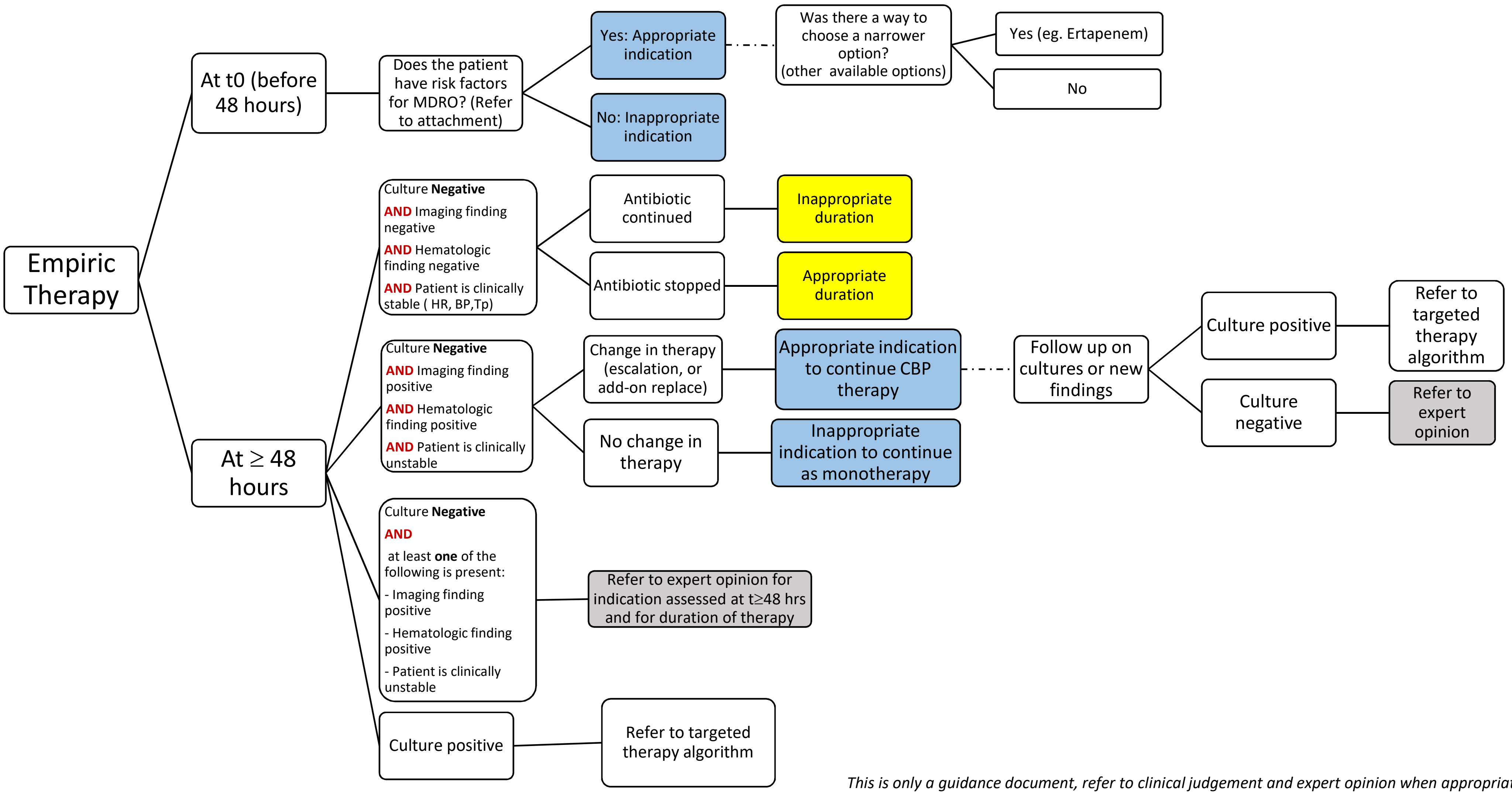

This is only a guidance document, refer to clinical judgement and expert opinion when appropriate

Targeted Carbapenem Therapy Algorithm (Positive Culture)

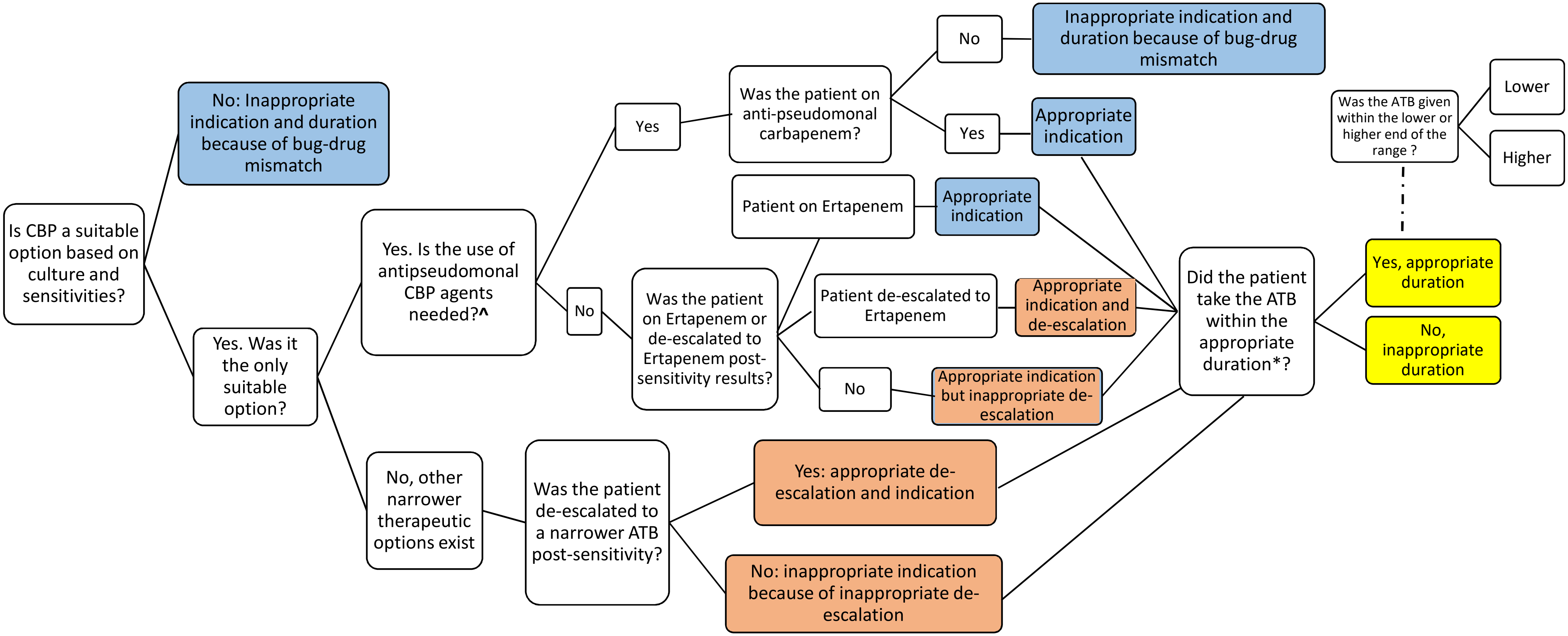

*: A duration is considered to be appropriate when it is between the recommended range including the lower and higher end of the range

^: Antipseudomonal agents (Meropenem/Imipenem-cilastatin) are indicated if the culture is positive for pseudomonas or those are the agents recommended for a specific indication

This is only a guidance document, refer to clinical judgement and expert opinion when appropriate

Surgical Prophylaxis Carbapenem Algorithm

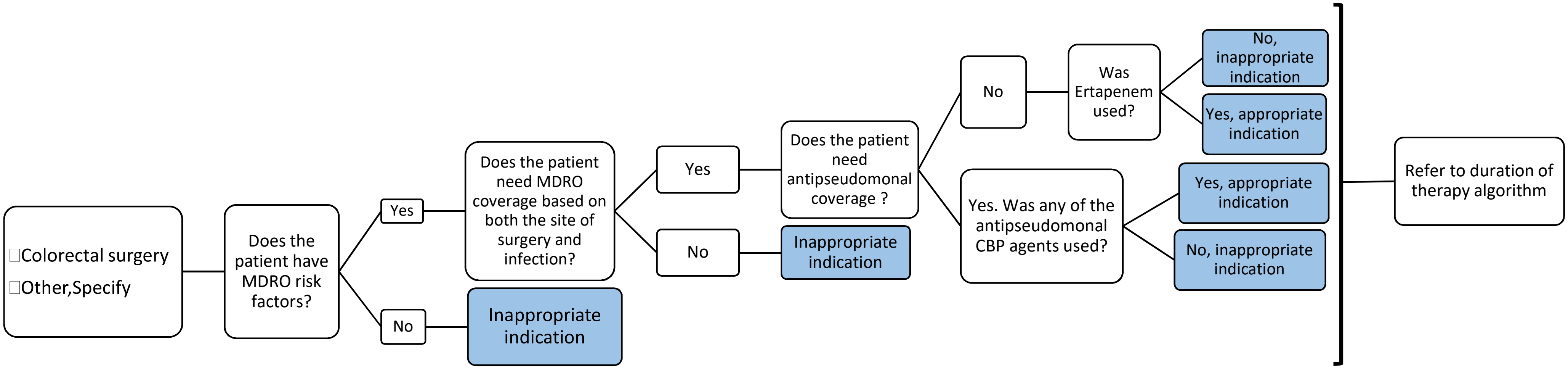

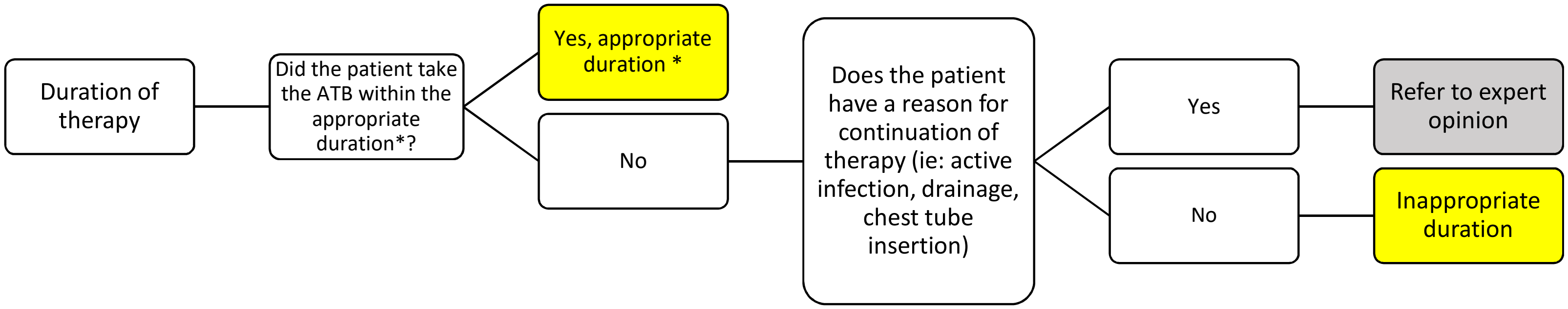

*: A duration is considered to be appropriate if the antibiotic was stopped 48 hours after a cardiovascular surgery or after 24 hours for all other surgeries.

This is only a guidance document, refer to clinical judgement and expert opinion when appropriate
