# Supplementary material for: Evaluation of Carbapenem Use Before and After Implementation of an Antimicrobial Stewardship-Led Carbapenem-Sparing Strategy in a Lebanese Tertiary Hospital: A Retrospective Study
Source: Front Cell Infect Microbiol. 2022 Mar 25;12:729491. doi: 10.3389/fcimb.2022.729491 (PMC8990088; doi:10.3389/fcimb.2022.729491)
Supplement: Supplementary file 2 [file DataSheet_2.pdf]

AUBMC

MDRO Risk Factors based on indication

<table>
<tr><td>Community Acquired Pneumonia: Pseudomonas risk factors</td><td>□Bronchiectasis Structural lung disease (chronic bronchitis, COPD, emphysema, interstitial lung disease, pulmonary fibrosis) AND taking chronic steroids/ history of repeated antimicrobial use
□Chronic systemic steroids
□Repeated antimicrobial use</td></tr>
<tr><td>HAP/VAP: MDRO risk factors</td><td>□Late onset—occurs after ≥5 days of hospitalization
□Antimicrobial therapy within the last 90 days (consider broad spectrum, multiple courses, etc.)
□Immunosuppressive disease and/or therapy
□ History of infection or colonization with a multidrug resistant organism
□ Chronic dialysis within 30 days</td></tr>
<tr><td>VAP: MDRO risk factors</td><td>□Antimicrobial therapy within the last 90 days (consider broad spectrum, multiple courses, etc.)
□Septic shock at the time of VAP
□ ARDS preceding VAP
□ 5 or more days prior to the occurrence of hospitalization
□ Acute renal replacement therapy before VAP</td></tr>
<tr><td>Catheter related blood-streaminfection:Pseudomonas Risk factors:</td><td>□Neutropenic
□septic patients
□Previously colonized with Pseudomonas Aeroguinosa</td></tr>
<tr><td>Febrile Neutropenia: Antipseudomonal beta-lactam monotherapy(including carbapenem)</td><td>□MASCC score (<21) High risk patients
Criteria below:
<table>
<tr><th>Characteristics</th><th>Score</th></tr>
<tr><td>Burden of febrile neutropenia with no or mild symptoms[a]</td><td>5</td></tr>
<tr><td>No hypotension (systolic blood pressure >90 mmHg)</td><td>5</td></tr>
<tr><td>No chronic obstructive pulmonary disease[b]</td><td>4</td></tr>
<tr><td>Solid tumor or hematologic malignancy with no previous fungal infection[c]</td><td>4</td></tr>
<tr><td>No dehydration requiring parenteral fluids</td><td>3</td></tr>
<tr><td>Burden of febrile neutropenia with moderate symptoms</td><td>3</td></tr>
<tr><td>Outpatient status</td><td>3</td></tr>
<tr><td>Age <60 years</td><td>2</td></tr>
</table></td></tr>
<tr><td>Diabetic foot infection: Pseudomonas risk factors:</td><td>□Residence in a warm climate
□High local prevalence
□History of pseudomonal infection
□Use of carbapenem is indicated Meropenem 1 g Q8h or imipenem/cilastatin 500 mg Q6h (use only when required; extended-spectrum β lactamase [ESBL]– producing pathogens expected)</td></tr>
<tr><td>UTI: Risk factors for MDRO</td><td>□Residence long-term care facility and presence of invasive devices
□Long term catheterization
□Broad spectrum antibiotics exposure within the past 90 days
□ History of MDRO
□ Recurrent UTI
□ Nosocomial UTI
□ History of ESBL producing agents within the past 60 days</td></tr>
<tr><td>Colorectal surgery</td><td>□Infected with MDR/ESBL
□Colonized with MDR/ESBL
□ Hospitalized for more than 7 days
□Multiple hospital admission within the past 3 months
□Antibiotic treatment of a systemic infection within the last month</td></tr>
</table>

References:

- IDSA guidelines (For each indication mentioned above)
- ASHP/ASP Pharmacist guide on the use of antimicrobial
- John Hopkins medicine on the empirical use of antibiotics
- IHS guidelines on the empiric use of antibiotics
- Antimicrobial prophylaxis on HIS
